# Challenges and Opportunities in the Statistical Analysis of Multiplex Immunofluorescence Data

**DOI:** 10.3390/cancers13123031

**Published:** 2021-06-17

**Authors:** Christopher M. Wilson, Oscar E. Ospina, Mary K. Townsend, Jonathan Nguyen, Carlos Moran Segura, Joellen M. Schildkraut, Shelley S. Tworoger, Lauren C. Peres, Brooke L. Fridley

**Affiliations:** 1 Department of Biostatistics and Bioinformatics, Moffitt Cancer Center, Tampa, FL 33612, USA; Christopher.Wilson@moffitt.org (C.M.W.); Oscar.Ospina@moffitt.org (O.E.O.); 2 Department of Cancer Epidemiology, Moffitt Cancer Center, Tampa, FL 33612, USA; Mary.Townsend@moffitt.org (M.K.T.); Shelley.Tworoger@moffitt.org (S.S.T.); Lauren.Peres@moffitt.org (L.C.P.); 3 Department of Pathology, Moffitt Cancer Center, Tampa, FL 33612, USA; Jonathan.Nguyen@moffitt.org (J.N.); Carlos.MoranSegura@moffitt.org (C.M.S.); 4 Department of Epidemiology, Emory University, Atlanta, GA 30322, USA; Joellen.M.Schildkraut@emory.edu

**Keywords:** digital pathology, cancer, tumor immune microenvironment, data science

## Abstract

**Simple Summary:**

Immune modulation is considered a hallmark of cancer initiation and progression, and has offered promising opportunities for therapeutic manipulation. Multiplex immunofluorescence (mIF) technology has enabled the tumor immune microenvironment (TIME) to be studied at an increased scale, in terms of both the number of markers and the number of samples. Another benefit of mIF technology is the ability to measure not only the abundance but also the spatial location of multiple cells types within a tissue sample simultaneously, allowing for assessment of the co-localization of different types of immune markers. Thus, the use of mIF technologies have enable researchers to characterize patient, clinical, and tumor characteristics in the hope of identifying patients whom might benefit from immunotherapy treatments. In this review we outline some of the challenges and opportunities in the statistical analyses of mIF data to study the TIME.

**Abstract:**

Immune modulation is considered a hallmark of cancer initiation and progression. The recent development of immunotherapies has ushered in a new era of cancer treatment. These therapeutics have led to revolutionary breakthroughs; however, the efficacy of immunotherapy has been modest and is often restricted to a subset of patients. Hence, identification of which cancer patients will benefit from immunotherapy is essential. Multiplex immunofluorescence (mIF) microscopy allows for the assessment and visualization of the tumor immune microenvironment (TIME). The data output following image and machine learning analyses for cell segmenting and phenotyping consists of the following information for each tumor sample: the number of positive cells for each marker and phenotype(s) of interest, number of total cells, percent of positive cells for each marker, and spatial locations for all measured cells. There are many challenges in the analysis of mIF data, including many tissue samples with zero positive cells or “zero-inflated” data, repeated measurements from multiple TMA cores or tissue slides per subject, and spatial analyses to determine the level of clustering and co-localization between the cell types in the TIME. In this review paper, we will discuss the challenges in the statistical analysis of mIF data and opportunities for further research.

## 1. Introduction

Characterization of a patient’s tumor immune microenvironment (TIME) is a topic that has stirred a great deal of interest with the advent of cancer immunotherapies [1,2]. Immunotherapies are agents that activate or act as a substitute for host antitumor immunity and have been revolutionary in the treatment of many cancers [3,4], including melanoma, lung cancer, and renal cell carcinoma [5]. However, the efficacy in other solid tumors has been modest and is often restricted to a subset of patients [2,6,7]. Clinical trials investigating immunotherapy often lack indicators of immune activity, making it difficult to discern the characteristics of the tumor immune microenvironment (TIME), which may have impeded response to therapy. Thus, immune profiling has become an important tool to aid in our understanding of immune checkpoints and to identify predictive markers of therapeutic response.

For example, tumors with a dense infiltrate of lymphocytes, also known as tumor infiltrating lymphocytes (TILs), are consistently associated with more favorable outcomes among cancer patients [1,8,9]. In colorectal cancer, a novel immunoscore was developed and validated based on the density of TILs (CD3+, CD8+) within the tumor and invasive margin [10,11,12]. This immunoscore was strongly associated with cancer outcomes, showing superior promise as a prognostic marker compared to stage and microsatellite instability status and has provided a powerful prognostic tool to improve outcomes of cancer patients [13,14,15,16].

The cellular composition of the TIME can be studied using many technologies and approaches, such as immunohistochemistry (IHC), multiplex IHC (mIHC) [17], gene expression deconvolution methods (CIBERSORT [18], xCELL [19]), single cell RNA sequencing (scRNAseq) [20,21,22,23], flow cytometry [24], and approaches for mass cytometry imaging (imaging mass cytometry [25,26,27] and multiplex ion beam imaging (MIBI) [28,29]). Conventional IHC is a widely used technique in the field of diagnostic pathology [30,31]. This technique takes advantage of the epitope–antibody interaction to show in situ protein expression or biomarkers on a formalin-fixed and paraffin-embedded (FFPE) tissue sample [32,33]. More recently, immune-profiling by IHC is becoming an important tool to predict immunotherapy response in various types of cancer [34,35]. However, the inability to identify more than 2–3 markers per slide limits the use of IHC to capture the complexity of immune phenotypes that exist in the tumor microenvironment [33,36].

The development of multiplex immunohistochemistry (mIHC) and multiplex immunofluorescence (mIF) has allowed for the assessment of multiple markers in a single experiment [37]. mIF/mIHC can be applied to both regions of interest (ROIs) of a whole tissue slide or to tissue microarrays (TMAs), thus simultaneously allowing for the study of the TIME in large number of samples. Another benefit of mIF platforms that they can detect both the abundance and spatial location within the tissue sample of multiple cell types. The use of mIF has been recently applied to study the spatial proximity between T and PD-L1 expressing cells in oropharyngeal squamous cell carcinoma [38], the spatial heterogeneity of macrophages in gastric cancer [39], and the spatial composition of myeloid cells in pancreatic cancer [40].

There are many challenges in the statistical analysis of data from mIF (or mIHC) studies. First, many studies involve multiple cores or ROIs from the same tumor tissue sample (i.e., repeated measurements). Second, when studying tumors that tend to have little immune infiltration or “cold”, often tumors have no positive cells for a marker of interest (i.e., zero-inflated data). To deal with this challenge, researchers often dichotomize the abundance measures into categories (i.e., no/low/high abundance). However, the challenge arises when defining the threshold to use in making these categories. Third, particularly in the case of TMA studies, there are often regions in which no cells were able to be measured (i.e., “holes” in the image). This uneven assessment of immune cells is often overlooked in spatial analyses, which often assumes that measurements are possible at all locations in the region of interest. Finally, batch effects between TMAs and phenotype misclassification (i.e., falsely calling a cell as positive for a marker) are common quality control issues related to mIF data. This review provides background on the analytic challenges related to mIF data, possible solutions and potential areas for future research. The concepts presented in this manuscript are illustrated using two large observational studies of ovarian cancer for which mIF data was recently generated (Nurses Health Studies I and II (NHSI [41,42], NHSII [43]), the African American Cancer Epidemiology Study (AACES) [44]).

## 2. Data Preprocessing and Quality Control of mIF Data

### 2.1. mIF Assay and Data Generation

**Figure 1:**
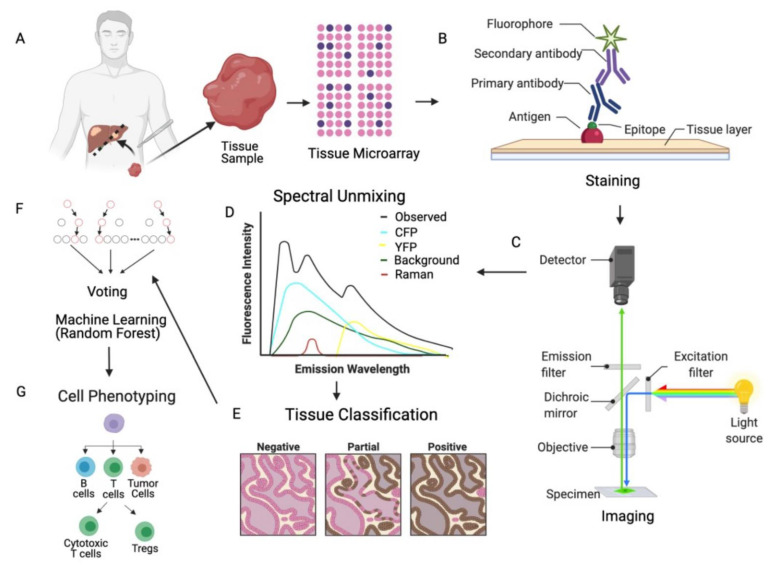
(**A**) Data are generated from biopsied tissue that is FFPE preserved, slices are then placed on a tissue microarray (TMA). (**B**) The slide is stained with antigen which are the sites that primary and secondary antibodies bind. (**C**) A range of different wavelengths of light is radiated at each location of the specimen and the wavelength emission goes through a spectral unmixing step (**D**), which deconvolves the observed intensity into cyan fluorescent protein (CFP), yellow fluorescent protein (YFP), background, and Raman components. In order to phenotype each cell (**E**–**G**), the tissue is segmented into tumor and stroma component using staining (**E**), intensities and information regarding the shape of the cell is used to derive the final phenotype via machine learning (random forest is a popular technique), followed by cell phenotyping (**G**).

Multiple platforms exist for mIF techniques, including standard IF scopes and multispectral technologies (Vectra 3.0^TM^/Polaris^TM^). The most important step is the selection of the primary antibodies to target the biomarkers of interest, with monoclonal antibodies often being used due to their high sensitivity and specificity [37]. These antibodies are then labeled with fluorophores which emit wavelengths that can be measured via microscope with a corresponding image saved for image processing and analysis. mIF technologies allow for the use of multiple antibodies to achieve the simultaneous detection of several marker on single tissue sample (recently nine or more markers). This technology has been used in research and clinical settings showing the utility of this approach for studying the TIME [45,46,47]. An overview of the data generation process with mIF technology is presented in Figure 1, focusing on cyclic-immunofluorescence and tyramide-based mIF. Cyclic-immunofluorescence requires sequential cycles where individual epitope or markers are labeled with antibodies, which is then followed by a signal amplification. Individual antibody complexes are then stripped after each round of antigen detection leaving the fluorophore covalently attached to a tyrosine residue of the target epitope and ready for the next round of immunofluorescence [48]. This is a labor-intensive procedure and may take several days to complete. However, fully automated staining protocols for mIF have been developed, saving time and improving staining variability [45,46,47,48,49].

Specialized cameras and software are needed to properly acquire multiple markers in a single image. Multispectral imaging (MSI) is the main technology used to accurately capture mIF images, whereby the intensity wavelength spectrum of every pixel is captured in the image [50]. This procedure generates a third dimension of information for every pixel in the image and potentially increases the number of wavelengths that can be captured from 4 bands to 10–30 bands (multispectral). The information from each multispectral image pixel is extracted to correctly separate all the captured wavelengths per pixel and acquire the desired image [48,51,52,53] using a spectral reference or spectral library. Once the spectral library is built, images are spectrally unmixed and image files with channel marker metadata are processed with MSI analysis [54,55,56,57,58]. One commonly used technology for mIF data generation is the Vectra 3.0^TM^/Polaris^TM^ system whereby images are processed within InForm [56,59,60,61] followed by analysis with the HALO Image Analysis Platform (Indica Labs, Albuquerque, NM, USA). In HALO, a supervised classifier using a random forest algorithm is trained to classify tissue as tumor, stroma, and glass (no tissue) regions [55,62,63]. Cell segmentation and marker quantitation is performed by compartmental examination of fluorescent intensity thresholds [60,61]. Analysis outputs are generated for each specific cell and summarized data per images including positive counts, co-localized phenotypes, marker intensity per compartment, percent of cells positive for a marker, and tissue area for density calculation and cell coordinates for spatial analysis [56,57,59,60].

### 2.2. Quality Control of Generated Data

#### 2.2.1. Conflicting Information between Markers (CD8 and FOXP3)

**Figure 2:**
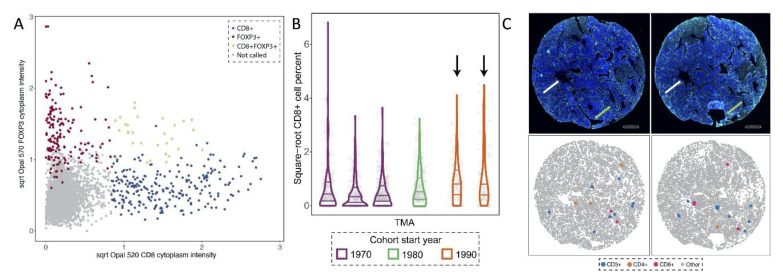
(**A**) Square-root transformed CD8 (Opal 520) and FOXP3 (Opal 570) fluorescence intensities of a tumor microarray core from an epithelial ovarian cancer tumor. Cell classifiers used in immunofluorescence studies can yield equivocal CD8+FOXP3+ assignments. Note that the CD8 threshold creates a clear separation of CD8+ cells, however the FOXP3 intensity threshold allows for a mixture of unassigned and FOXP3+ cells. (**B**) Square-root transformed percent of CD8+ cells detected in 1312 epithelial ovarian cancer tumor slices from 445 participants. The tumor slices come from 6 different TMAs, with initial collection of tissues starting at different times since the 1970s. The three horizontal lines represent the 1st, 2nd, and 3rd quartiles, and the width of the violin plots represent the number of slices showing a given percentage. As showed by narrower violin bases, the TMAs generated starting in the 1990s show less zeroes in CD8+ cell counts compared to the other TMAs generated in previous years. (**C**) mIF images from the same core from an ovarian cancer TMA. The two slices were stained with pan-cytokeratin (PCK) but were applied two different mIF panels to detect B and T cells (top). The cells detected after image processing are shown. Differences are observed between the two slices, including the presence of “holes”, making difficult to perform comparative spatial analysis of the two slices from the same TMA core. The white arrows correspond to a region that is similar across the different sections of the same core, while the green arrows correspond to regions that are dramatically different. Illustration that plots generated from mIF data capture these features and maintain the cell locations (bottom).

In the process of cell phenotyping (i.e., calling a cell as positive for a marker), there are often situations in which cells are misclassified or mis-phenotyped. For example, in designing mIF assays, CD3 is often used as a general T-cell marker with additional markers added to distinguish between types of T-cells, with CD8 often used as a marker for cytotoxic T-cells and FOXP3 used as a marker for regulatory T-cells [64]. In the cell phenotyping, a machine learning algorithm is applied to the intensity data (i.e., machine learning algorithm within the HALO software) resulting in cells being called positive for a marker [56,65]. This approach may lead to equivocal cell type assignments, as illustrated in Figure 2A. In this case where cells were called positive for CD3, CD8 and FOXP3, one option would be to classify the cells as only a T-cell (CD3+) and remove the conflicting assignment of cytotoxic T cell (CD8+) and regulatory T cell (FOXP3+). Another option would be to apply a different cell phenotyping algorithm to improve cell phenotype assignments, such as a supervised approach in which the highest intensity from a group of known false positives is used as the threshold for a given marker [66]. Finally, newer approaches have been developed to assist cell annotations, such as the CITE-Seq atlases [67].

#### 2.2.2. Batch Effects

Minimizing sources of assay variability (e.g., technical variation) is important for scientific validity, reproducibility and to maximize statistical power [68]. Sources of variability for mIF assays may include batch-to-batch variations across TMAs or a large set of whole slide ROIs and inconsistent quality of staining related to tissue characteristics, such as age of the tissue samples (e.g., if collected over years or decades). Therefore, after summary data have been generated for a set of samples (e.g., percent or density of positive cells), it is recommended that a quality control step be completed to compare the distribution of positive cells across subsets of samples defined by TMA, timing of staining or other technical “batch” factors, and/or tissue characteristics (e.g., histology, grade). Visual plots of the percent positivity, such as box plots, violin plots, and scatter plots, in each subset are useful tools for these qualitative quality control checks (Figure 2B). A binary positivity measure (e.g., percent of cases with >5% positive cells) could also be calculated. If certain tumor characteristics are associated with the prevalence of the cell type of interest and vary across sample subsets, these quality control checks should be conducted among similar samples (e.g., among cases with the same tumor histology).

If the quality control checks reveal unexpected variability, the issues may be addressed by staining a new slide (or slides), returning to the image analysis stage, or conducting additional analyses during the statistical analysis. For example, examining distributions of markers across TMAs might reveal certain TMAs with much higher percentages of positive cases than others; this might be due to higher levels of background staining in those TMAs, which can be addressed by adjusting the image analysis. For a factor that cannot be altered, such as sample age, the analysis could adjust for this batch effect in the statistical model or conduct the analyses separately among the different subgroups of the factor of interest to examine whether the findings are consistent across subgroups. It is recommended that all cores from the same tumor be included on the same TMA so that the batch effect is not confounded with the within patient variability. It is also recommended when constructing TMAs for mIF studies that the use of randomization be done to ensure TMAs are balanced in terms of any relevant clinical (i.e., stage or tumor histology) and/or technical factor (i.e., age of tissue sample).

One of the most appealing features of immunofluorescence is the ability to assess numerous markers simultaneously, which allows for discrimination of a variety of cell types. Nonetheless, different panels of markers are applied to sequential tissue sections where the location of cells are not consistent. This use of sequential tissue sections makes different mIF panels not comparable in terms of the spatial organization of cells thus limiting the ability to study the colocalization of markers on different panels. Careful sequential cutting of the tissue to avoid rips or folding, while preserving orientation, requires a highly skilled and experienced technician [69], and it partially overcomes the challenge as cellular heterogeneity among sequential sections is expected (Figure 2C). It has been shown that different panels can be applied to the same tissue section, however, appropriate technical controls need to be carefully implemented to assess staining errors [53], and masking effects from previous markers can occur [70]. Consequently, spatial analysis needs to be conducted and interpreted separately by marker panel.

## 3. Analysis of Summary Data

### 3.1. Analysis of the Number, Percentage or Density of Cells Positive for Immune Marker

**Figure 3:**
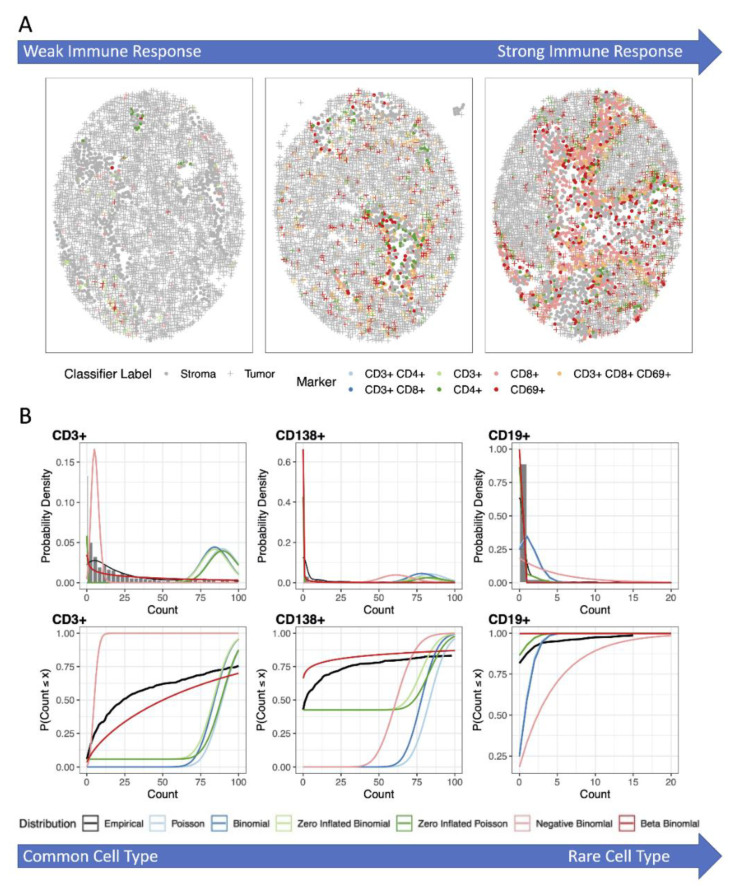
(**A**) Illustration of the possible differences in immune activity within TMA cores. (**B**) Histograms with empirical and theoretical probability density function (top) and empirical and theoretical cumulative probability distribution (bottom) to guide in the selection of modelling assumptions for markers becoming increasingly rare (from left to right). The Poisson and binomial distribution do not account for overdispersion or zero-inflation, negative binomial and beta-binomial only account for over dispersion, and zero-inflated Poisson and binomial distribution only account for zero-inflation. The negative binomial and beta-binomial distributions are suitable for cell types where less than 50% of the cores have 0 for that cell type (CD3+, CD138+), while zero inflated models are best for excess 0s (CD19+).

Frequently, the analysis of interest involves the extraction of summary statistics (i.e., does the abundance of PD-L1 positive cells differ between responders and non-responders to an immunotherapy). These summary statistics are calculated for each of the cell phenotypes and offer a general picture of the abundance across the tissue sample. Common summary statistics include the number, percentage, and the density of positive cells expressing a given marker. These metrics can be calculated manually, although higher accuracy and efficiency can be achieved by using image analysis software (e.g., HALO) [71]. To account for TMA or ROI size, the percentage of positive cells out of a total number of measured cells and/or counts per unit of area (density) are widely used. These summary measures can be computed separately for the tumor and stromal compartments [72]. When measuring immune cells in “cold tumors”, the total number of immune cells is expected to be a very small proportion of the total number of cells, possibly leading to apparently small between person variation (Figure 3A). For especially rare immune cell subsets, an alternative approach would be to look at the percent of a type of immune cell out of only the immune cells, not out of the tumor and stroma cells (e.g., number of CD8+CD3+ cells out of total number of CD3+ cells). These summary measures are then used in statistical models to determine associations between immune markers and various outcomes. However, care in picking the statistical modeling approach is needed as often these summary measurements do not follow a normal distribution.

Many statistical analyses approaches are based in Gaussian or normal distribution theory for parameter estimation (confidence intervals) and hypothesis testing (*p*-value). For the analysis of the number of positive cells ($X_{i}$) out of the total number of cells measured ($N_{i})$ for sample *i,* the distribution for the analysis is a binomial distribution (i.e., $X_{i}\sim BIN\left(N_{i},\;p_{i}\right)$). If the probability of being a positive cell ($p_{i})$ in the sample is low (i.e., “rare event” or “cold” tumor) and assuming the number of cells $(N_{i}$) is large, the binomial distribution can be approximated with a Poisson distribution (i.e., $X_{i}\sim POI\left(\lambda_{i}=N_{i}p_{i}\right)$). In these distributional settings, a generalized linear model can be used to assess the association of a predictor variable on the number of positive cells for a given marker (i.e., does the level of cell positivity differ between treatment groups) [73,74].

Another approach often used is to model the percentage of positive cells ($X_{i}/N_{i})\;$ or the density using normal theory methods (i.e., two-sample t-tests, linear regression, ANOVA) or with the corresponding non-parametric method (i.e., Wilcoxon rank-sum test or Mann–Whitney U test). Prior to using the normal theory methods, a plot of the percentages or densities by the predictor variable (i.e., treatment group) as well as a plot of the residuals from the statistical model should be conducted to determine if the normal distributional assumption is reasonable [75,76]. As the percentage is bound between 0% and 100% and for immune cells, many samples have few cells positive for the marker of interest, a left skewed distribution or “pile-up” of observations by 0% (Figure 3B) is typical. In this case, a suitable transformation to the percent or density, such as a natural log (ln), logit (i.e., log(*p*/1 − *p*)), or square-root transformation, should be applied. Note that in the setting in which the dataset contains 0% (or 100%), only a square-root transformation is possible.

Alternatively, the continuous measure of abundance (e.g., percentage positive, density) can be categorized into groups. The challenge with this approach is the selection of the threshold used to make the categories. Often, researchers use the median (50th percentile) or the quartiles (25%, 50%, and 75%) of the values in a dataset to construct the various groups/categories. In the context of biomarkers, often a biologically relevant threshold is selected. For example, in breast cancer, the estrogen receptor (ER) must be expressed in more than 1% of cells to be called ER+ [77]. One challenge is determining the clinically and biologically relevant thresholds for “positivity”. For example, it is difficult to compare results between clinical trials for PD-1/PD-L1 as studies have used different thresholds for determining who received benefit from immunotherapies, with some trials using a threshold of 1% positivity in tumor cells [78], while other studies have used a threshold of 10% [79]. This threshold for PD-L1 “positivity” can also differ from 1% to 50% in different cancer types due different biological and immune mechanisms [80,81,82], with little research presented on thresholds for other immune markers, such as cytotoxic T-cells.

An alternative approach for setting the pre-defined threshold is to determine the “optimal cut-point”, which is selected to maximize the test statistic of interest [83,84]. From a statistical standpoint, the optimal cut-point approach is “data-snooping” since the results from the statistical test inform the creation of the thresholds [85]. On the other hand, for discovery and hypothesis generation purposes, it is clinically and biologically useful to determine an optimal cut-point that can be used and validated in other studies. However, a challenge in determining optimal cut-points is that the number of samples in a group/category can get very small when categorizing across multiple variables. In a study of the TIME of ovarian cancer in African American women, the optimal cut-points as related to overall survival for dichotomizing CD3+, CD3+CD8+ and CD3+FOXP3+ ranged from 1–6%, restricting groups to have at least 10% of the sample size [86].

### 3.2. Analysis Using Zero-Inflated and Over-Dispersed Distributions

An additional challenge in the analysis of summary data from mIF studies are scenarios in which many of the samples have no positive cells for a given biomarker of interest (i.e., zero-inflated distribution; Figure 3B). This is especially true for “immune cold” tumors where only a few cells express the immune markers of interest. In recent decades, extensive research has been conducted in zero-inflated statistical modeling, mainly for a Poisson distribution [87], referred to as a “ZIP” model. Zero-inflated models have been recently extended to the analysis of microbiome or metagenomics data, either in the context of Poisson [88], negative binomial [89,90], or beta-binomial [91] distributions. These models have also been extended with random effects to account for repeated measurements or dependency in observations [87,88,92].

In general, the zero-inflated models are statistical methods that allow for frequent zero-valued observations or “overdispersion”. These models are considered a type of “mixture model” or “two-part model” where the model involves a mixture of a standard distribution (i.e., Poisson) and a point mass distribution at 0. Assuming the count of positive cells follows a Poisson distribution (as an approximation to the binomial distribution), another approach to account for overdispersion is with a negative binomial distribution. For example, in the context of RNA-sequencing data analysis, the read counts from high-throughput sequencing often are assumed to follow a negative binomial distribution instead of a Poisson distribution to account for the overdispersion in the data (i.e., the variance in gene expression abundances is much larger than the mean abundances, a departure for a Poisson distribution which assumes the mean equals the variance) [93,94,95]. Another option to account for the zero-inflation would be to model the data with a beta-binomial distribution. The beta-binomial distribution is the binomial distribution, $X\sim Bin\left(N,\;p\right),\;$ in which the probability *p* of being a cell positive for an immune marker out of *N* cells is not fixed but rather a random variable with $p\sim Beta\left(\alpha ,\;\beta \right)$. The beta-binomial distribution has been extensively used in biological and medical research [96,97,98,99,100,101], frequently within a Bayesian framework [102]. As no one distribution will fit all markers in a study the best, it is recommended to assess model fit for the various distributions at the beginning of the analysis and select the appropriate model for each marker of interest, thus providing the highest power to detect true associations (Figure 3B). In our experience, the zero-inflated binomial models often fit best when there is a larger percentage of 0 positive cells counts while the beta-binomial works well when the level of zero counts is not as extreme.

### 3.3. Repeated Measurements

mIF studies often include multiple tissue samples from the same tumor (i.e., multiple cores in the case of TMAs or regions of interest (ROIs) in the case of whole slides). This aspect of the data requires the application of statistical analysis methods that account for dependency in measurements taken from the same subject. Often, this is completed via the use of mixed- or random-effect modeling [103] within the context of a linear model (i.e., linear regression using a normal distribution) or generalized linear model (i.e., logistic regression using a binomial distribution). The challenge in fitting these mixed models, particularly in the case of a generalized linear mixed model, is that they can be computationally intensive and require numerical approaches for parameter estimation and hypothesis testing, such as use of the expectation–maximization (EM) algorithm [104], Newton–Raphson method [105], numerical and Gaussian quadrature, or Markov chain Monte Carlo (MCMC) [106,107]. Hence, one of the key steps in fitting mixed models is checking for convergence of the estimation algorithm.

Alternatively, if one is not able to fit these more sophisticated mixed models, the mean of the density or percent positivity across the samples from the same subject can be used. However, this approach may under-estimate the variability in the measurements (except in cases where the repeated values for a subject are very consistent) and hence could impact the statistical inference (i.e., the type I error rate will be increased and the confidence intervals will be too small [108]). However, for very small studies, this might be the only approach possible. Lastly, if you assume the density or the percentage of positive cells follows a normal distribution and are interested in whether these values differ between two or more groups, a repeated measures ANOVA analysis could be completed; noting that unlike mixed models, additional covariates or predictors cannot be included in the model [109].

## 4. Clustering and Cooccurrence in Spatial Analysis of mIF

Research in ecological and spatial statistics over the last 40 years have developed many analytical methods that can be leveraged for studying the spatial architecture of the TIME. As such, many of these methods have been recently applied to the analysis of mIF data (Table 1). In general, these methods can be applied at the pixel/region-level or the cell-level and can be classified into three collections: count based methods at the pixel or region-level; point process methods at the cell-level; and distance-based methods at the cell-level. The pixel or region-level methods typically quantify a measure of interest for each pixel, followed by assessment of the variation in measurements in order to study the diversity, heterogeneity or autocorrelation across the entire image. On the other hand, cell-level methods often involve studying the nearest neighbor of each individual cell or the distance between cells to quantify the degree of clustering, cooccurrence, or segregation of cell populations.

**Figure 4:**
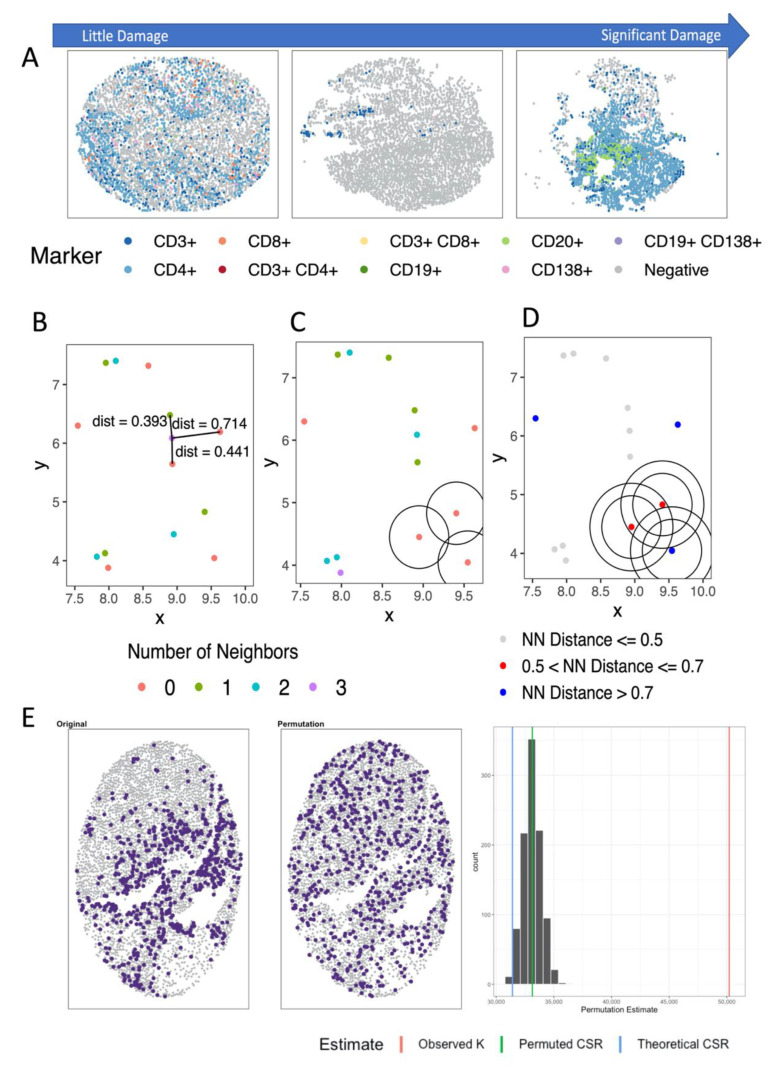
(**A**) Example images showing cores with little to significant damage. A point process generated from simulated data illustrating different approaches for spatial analysis: (**B**) Distance- or nearest neighbor-based methods, (**C**) neighborhood methods such as $K\left(r\right)$, $\;L\left(r\right),$ and $\;M\left(r\right)$; and (**D**) distance to neighbor measures such as $\;h\left(r\right)$ and $g\left(r\right)$**.** (**E**) Example of original (left) and permuted point process (middle) with resulting histogram (right) of permutation-based estimates of *K* showing difference in theoretical and permuted-based estimates of CSR where the theoretical value under-estimated the value of  K under CSR.

There are challenges in using these co-localization or clustering measures directly due to image curation issues (i.e., “holes” or areas in the image with no cells measured particularly in the setting of TMA studies) (Figure 4A) and/or studying rare cell types (large proportion of samples with 0 positive cells) (Figure 3A), which may lead to departures from the underlying assumptions for which the statistics were developed. In order to relate co-localization or clustering of immune cells to a phenotype (i.e., survival, treatment response), it is also necessary to ensure that data collected across multiple samples is comparable by (1) normalizing measurements across samples; (2) correcting the estimate to account for regions in the image in which no cells were able to be measured; (3) correcting for edge/border effects; and lastly (4) accounting for the correlation between abundance and spatial measures (i.e., not able to estimate spatial clustering when the abundance is close to 0%). These steps are critical to ensure the magnitude of features have the same meaning across the TMA cores or ROIs.

One way to account for many of these issues is with the use of permutation or Monte-Carlo methods, where the status (i.e., positive for immune marker) of independent blocks of pixels or cell is permuted, thus providing a broad set of scenarios to help evaluate the significance of the observed pattern in the image [125]. Using permutations, image specific distributions of the statistic of interest are estimated under the assumption of complete spatial randomness (CSR) (i.e., null distribution). The mean of this estimated distribution under CSR can then be used to normalize the estimate for each sample, thus producing a measure of the “degree of spatial clustering or colocalization” (i.e., observed spatial statistics—the mean of the empirical distribution of the statistic under the assumption of CSR) (Figure 4E). The association analyses would be based on this degree of clustering statistic and an adjustment for the overall abundance of the marker in determining the involvement of spatial co-localization/clustering on the phenotype of interest (i.e., response to immunotherapy, overall survival, tumor grade). The permutation of independent blocks of pixels or cell locations can provide a broad set of scenarios to help evaluate the significance of the observed pattern in the image, allowing for assessments separately in the tumor and stroma compartments of the tumor [125].

### 4.1. Pixel or Region-Based Methods

The first type of methods used in studying the spatial contexture of the TIME are pixel- or region-based methods. These methods involve splitting a TMA core or ROI into separate non-overlapping regions where a summary measure of two cell types can be computed (e.g., number of positive cells; number of cell types observed in region). One such method is the Morisita–Horn (MH) index, which is a measure of spatial dispersion of individual populations of interest (i.e., immune cell populations) [110,111]. The MH index was recently used in a study of HER2+ breast cancer, in which a high degree of colocalization between immune and tumor cells was associated with a higher probability of 10-year survival [126,127]. However, a limitation of the MH index is that this measure does not provide a reliable estimate when one of the immune cell populations is rare, with methods proposed to reduce the under-sampling bias [128]. In contrast, the Duncan segregation index [113], developed in the context of gender based occupational segregation, can also be used as a region-based segregation or co-localization measure to determines if the proportion of immune cell populations in a region (i.e., CD3+CD8+ cells vs. CD3+FOXP3+ cells) differ from the expectation under no co-localization. However, neither MH index nor Duncan’s segregation index account for the spatial autocorrelation between neighboring regions that is known to be the case with cellular processes, such as cell signaling. Cell signaling is one mechanism by which T cells are regulated, and it has been discovered that CD4 and T-cell antigen receptor (TCR) cells tend to form separate clusters in protein islands while CD4 and TCR cluster together upon T cell activation [129].

In contrast to the MH and Duncan segregation index developed for ecological and social science research, the Voronoi diagram [130] was developed in the field of mathematics for the study of quadratic forms and has been used extensively in geophysics and meteorology for spatial analysis. In terms of application to study of the TIME, a variation of Voronoi diagrams was used to measure “cell sociology” in lung adenocarcinoma [131]. Using the neighbors defined by the Voronoi diagram, cell-cell interaction measures were computed based on “adjacency” with permutations to obtain an image specific null distribution. This method was used to reveal that high T-cell clustering is associated with lack of recurrence. Additionally, in non-recurrent cases, a higher frequency of tumor cells with neighboring CD3+CD8- cells were observed than expected by chance [131].

### 4.2. Distance- and Nearest Neighbor-Based Methods

One of the most common methods to study the co-localization and cooccurrence of immune cells in the TIME is nearest neighbor distance (NND) [60,132,133]. This approach can be used to determine which immune cell type tends to cluster close to tumor cells by computing the distance (e.g., Euclidean) between the immune cell and closest tumor cell (Figure 4B). NND was used in a study of pancreatic ductal carcinoma where it was observed that myeloid cells (CD16+) were closer to tumor cells than T and B cells [134]. In melanoma, NND analysis showed that proximity of cancer cells to cytotoxic lymphocytes depends on the expression of Ki67 (tumor cell proliferation marker) and that the expression of HLA-DLR (macrophage activation marker) impacts the proximity of macrophages to cytotoxic lymphocytes [132]. However, careful attention to the implementation of this approach is needed when large “holes” or regions are present in TMAs where cells are not able to be measured. To overcome this challenge, permutation or Monte-Carlo methods can be used, as outlined in the next section.

### 4.3. Spatial Point Process Based Methods

The final type of spatial analysis methods for studying the TIME are those that are based on spatial point processes. In this setting, the locations of the immune cells in the TIME can be thought of as a spatial point process, a random pattern of points in a predefined area or “window”. The simplest point process is a homogeneous or stationary Poisson point process, where the rate of an immune cell is constant over the entire region of interest [116,135,136]. However, in studying the TIME, we often wish to know if the arrangement of cells positive for an immune marker fail to meet the assumption of CSR for homogenous spatial processes. Attraction (clustering), repulsion and competition (dispersion) are examples of interactions that would lead to violation of CSR.

Analysis of spatial Poisson point processes can consist of studying the number and location of points within a certain region, following a Poisson distribution. An alternate way to study spatial Poisson point processes is in terms of studying the spacing of events (distance to nearest neighbor) to understand how events are clustered, where the distance of the nearest neighbor follows an exponential distribution. Several quantities will be described in the following sections (Table 1), but it is important to remember that these concepts are complementary.

#### 4.3.1. Analyzing Number of Neighbors

Clustering and cooccurrence can be studied directly by analyzing the location of cells themselves. This requires defining a neighborhood as circle, with a specified radius *r*, surrounding a cell and defining each cell within the neighborhood (Figure 4C). These methods can be applied to questions related to a single cell type or two cell types with the assumption that the underlying point process follows CSR. A popular quantity that studies the number of nearest neighbors and has been used in ecological statistics for the clustering of objects (i.e., trees) is Ripley’s *K*(*r*) [115,116], where *K*(*r*) is expected to increase quadratically with respect to *r*. Besag introduced a modification to *K*(*r*), *L*(*r*)*,* which theoretically increases linearly with *r* [117]. The degree of clustering is estimated as the difference between the observed estimate and the estimate under CSR, where a positive difference indicates clustering and a negative distance indicates dispersion. Marcon also proposed a modification to *K*(*r*), referred to as *M*(*r*), where the expected value is 1 for all values of *r* and can be interpreted as the percent of clustering/repulsion observed [118]. Similarly in concept to *K*(*r*) and increases quadratically with respect to *r*, the hypothesized Interaction Distribution (HID) has been proposed to measure the interaction or co-localization between immune cells [121]. However, the HID does not incorporate edge corrections or centers the observed quantity about the expected value. The application of HID to oropharyngeal squamous cell carcinomas found that high co-clustering of CD8+ and PDL1+ as well as CD8+, PD1+ and PDL1+ was associated with worse survival [38].

#### 4.3.2. Analyzing Distance to Neighbor

The quantities in this section have a different interpretation than the neighborhood-based measures. They are interpreted as probabilities of an event occurring within a radius *r*. The nearest neighbor distance distribution (“event-to-event” distribution) is denoted by *G*(*r*) [116]. A counterpart to *G*(*r*) is the empty space function, *F*(*r*) [122], or often referred to as the spherical contract distribution (SCD), which is estimated by selecting arbitrary locations as opposed to the search region being centered at each cell. Similar to *K*(*r*), it is computed as a variety of radius values, *r,* and can be compared to the estimate under CSR by direct comparision to the theoretical distribution or computing $J\left(r\right)$. Bivariate versions of *F*(*r*) and *G*(*r*) have been derived to study the spacing between two cell types and has been used as a surrogate for immune cell infiltration of a tumor, where interactions between T-regulatory cells (Tregs, CD3+FOXP3+) and tumor cells, and cytotoxic (CD3+CD8+) and Tregs were shown to improve survival in patients with NSCLC lung cancer [137].

Finally, since the *G(r)* is the cumulative distribution function (CDF) of an exponential random variable, a hazard function can be estimated with the interpretation as the chance the nearest neighbor is within a small ring (Figure 4D). The pair correlation function or radial distribution function, *g*(*r*) and *J*(*r*)*,* respectively, and the spherical contact distribution (SCD) or *F*(*r*) were used to study CD68+ macrophages in human head and neck tumors [119]. In this study, they found that the level of clustering of CD68+ cells (macrophages) is related to the level of leukocyte infiltration in the sampled ROI. The function *g*(*r*) describes how the density of cells differs as a function of distance *r* from a reference cell [120] and has been used extensively in the field of physics [138], while *J*(*r*) involves the ratio of 1 − *G*(*r*) to 1 − *F*(*r*) and its expectation is 1 under CSR [124].

#### 4.3.3. Considerations

One challenge in using point process methods is the correction for edge effects. An edge effect arises as cells on the border lose neighboring cells that are located outside the sampled region. Corrections for edge effects have been developed for many methods, including Ripley’s *K*(*r*). Two common adjustments are isotropic and translation corrections [135,139]. These corrections are necessary as the point process continues outside of the study region; hence analysis using only the cells measured in the image will underestimate the number of cells in the proximity for cells near the border. An excellent demonstration and explanation of edge corrections are in Gabriel et al. [140].

Another issue in using point process methods is that they often involve functions computed at a variety of radii values (*r*) producing a function or curve. One approach would be to select a value of r to use to estimate the spatial measures that would then be used in the association with the phenotype of interest. Alternatively, the spatial measures could be treated as all the values of *r* as a function or curve and complete functional data analysis (FDA). FDA is the analysis of information in curves [141]. One approach would be to compute the area between the empirically observed function/curve, where the curve would be computed under the assumption of CSR. This area, as opposed to the curve, can be used for the association analysis. This approach was recently used to characterize the immune landscape of malignant pleural mesothelioma tumors into two distinct patterns related to the clustering of tumor-associate immune cells [133].

We have applied Ripley’s *K*(*r*) to the context of ovarian cancer, where patients with tumors having high abundance and a low degree of spatial clustering of CD3+CD8+ cells had significantly better survival compared to the patients with high spatial clustering [86]. A permutation approach was used to estimate the distribution of *K*(*r*) under CSR in order to correct for the potential bias in measurements due to “holes” in the TMA cores (Figure 4E). Additionally, edge effects were corrected and with the use of permutation or Monte-Carlo methods, clustering was assessed in the tumor and stroma components separately.

## 5. Discussion and Conclusions

In this review paper, an overview was provided of the statistical challenges and opportunities in the analysis of mIF data ranging from data quality control and assessment of batch effects to the statistical analysis with a zero-inflated distribution within a repeated measurements context. The mIF technology allows for an in-depth analysis of the tumor immune microenvironment (TIME) with assessments of both the abundance and spatial location of immune cells in the tumor microenvironment. The described analytical approaches are also applicable for the analysis of data from other technologies used to study the TIME (i.e., mIHC [17,37] and image mass cytometry [28]). Additionally, we addressed challenges of analyzing data from both TMAs and ROIs. In particular, TMAs tend to have smaller tissue areas (i.e., fewer cells measured) and areas that are not able to be assessed (i.e., “holes” in the image).

The presented analysis methods assumed that cell phenotyping of tissue specimens had been completed by a machine learning algorithm (i.e., converting the intensity measurement to a binary call of positive or negative for a cell type marker). Another approach would be to complete the statistical analysis on the raw intensity data, thus avoiding the issue of cell misclassification. However, care is needed as it is not clear if the intensity values have any intrinsic meaning. Additionally, batch effects in the intensity values are possible and additional analysis is needed to normalize and correct for batch effects in the samples [142,143,144].

For the analysis of the summary measures, it is recommended to assess model fit to determine the appropriate testing framework (i.e., use of zero-inflated or over-dispersed distribution) and if possible, to model using a count-based distribution (i.e., binomial distribution). In the context of survival analysis where the focus is assessing the association of the abundance of an immune marker with a time to event endpoint (i.e. overall survival, time to progression on immunotherapy), care is needed in selecting the modeling approach. When there are many samples with zero positive cells and/or a very skewed distribution, the abundance measure (i.e., percentage, density) should be categorized based on biologically relevant cut-points to aid in the interpretation and comparison of results across studies.

A benefit of using mIF technologies is the generation of spatial information for each cell. In studying the spatial architecture of the TIME in many samples/subjects, the spatial measurements should be comparable across tumor samples (i.e., normalization). Additionally, in the case of TMAs, approaches to account for potential biases due to “holes” or regions in the image where cells were not able to be measured should be used, including permutation or Monte-Carlo methods. The mean of the empirical distribution computed under the assumption of spatial randomness can be used to compare to the observed value, producing an estimate of the degree of spatial clustering.

Lastly, further research is needed to develop methods and approaches that can combine information on the spatial TIME generated by mIF methods with other complementary methods used for studying the TIME (i.e., single-cell RNA-seq [22,23,145]; spatial transcriptomics [146,147,148]). Additionally, approaches that are able to determine tumor immune subtypes (i.e., “cold” vs. “hot” tumors, intratumoral immune states [149]) or “immunoscores” [11,13], using both abundance and spatial architecture of the TIME, are needed. Particularly these new approaches should accommodate for the zero-inflated/over-dispersed nature of the measurements within a repeated measurements analysis framework.

In conclusion, mIF technology has enabled the TIME to be studied at an increased scale, in terms of both the number of markers and the number of samples in a cost-effective manner. Another benefit of mIF technology is the ability to measure not only the abundance but also the spatial location of multiple cells types within a tissue sample simultaneously. This allows for the study of the co-localization and co-clustering of different types of immune markers simultaneously within both the tumor and stroma compartments. The development of methods and approaches that can deeply characterize the spatial heterogeneity of the TIME, classify immuno-phenotypes and identify immuno-spatial patterns could inform novel immunotherapy treatment approaches, as well as immunological biomarkers that can be used to predict immunotherapy response.

## Figures and Tables

**Table 1:** Summary of the spatial measures outlined in Section 4 with the distinction for the spatial point processes being made to highlight the duality between distance to the nearest neighbor and locations of events.

Type of Analysis	Name	Empirical Formula	Theoretical Value under CSR	Comments
Pixel/Area Based	Morisita Horn Index [110,111]	$MH\left(p_{1},p_{2}\right)=\frac{2p_{1}p_{2}}{p_{1}^{2}+p_{2}^{2}}$ =$\frac{2\sum_{k=1}^{P}p_{1}^{k}\times p_{2}^{k}}{\sum_{k=1}^{P}{\left(p_{1}^{k}\right)}^{2}+\sum_{k=1}^{P}{\left(p_{2}^{k}\right)}^{2}\;}$		Robust to settings involving small number of cells [112]
	Duncan Segregation Index [113]	$D=2^{-1}\overset{P}{\underset{k=1}{\sum }}|p_{1}^{k}/p_{1}-p_{2}^{k}/p_{2}|$		Do not work well for rare cell populations Checkerboard Problem [114]
Nearest Neighbor	Euclidean Distance	$d\left(c_{i},c_{j}\right)=\sqrt{{\left(x_{i}-x_{j}\right)}^{2}+{\left(y_{i}-y_{j}\right)}^{2}}$	${\left(\lambda \pi r^{2}\right)}^{-1}$	
	Nearest Neighbor	$\underset{j}{\min}d\left(c_{i},c_{j}\right)$	${\left(\left(n-1\right)\lambda \pi r^{2}\right)}^{-1}$	
Spatial Point Processes	Ripley’s K [115]	$\hat{K}\left(r\right)={\left(n\left(n-1\right)\right)}^{-1}\overset{n}{\underset{i=1}{\sum }}\underset{i\neq j}{\sum }1\left(d\left(c_{i},c_{j}\right)\leq r\right)e_{ij}$	$\pi \;r^{2}$	Summarizes larger scale than G Can be modified to handle non-homogeneous spatial processes Cumulative and no information on what radius the clustering occurs Better overall performance than F, G, J [116]
	Besag’s L [117]	$\hat{L}=\sqrt{\frac{\hat{K}\left(r\right)}{\pi }}$	r	
	Marcon’s M [118]	$\hat{M}=\frac{\hat{K}\left(r\right)}{\pi r^{2}}$	1	
	Pairwise Correlation Function [119,120]	$\hat{g}\left(r\right)\;$ $={\left(2\pi \right)}^{-1}\overset{n}{\underset{i=1}{\sum }}\underset{i\neq j}{\sum }\frac{\kappa \left(r-d\left(c_{i},c_{j}\right)\right)}{d\left(c_{i},c_{j}\right)}e_{ij}$	$\;\frac{K^{\prime }\left(r\right)}{2\pi r\;}$	Not cumulative Interpreted as the probability two cells are a specified distance apart Best suited for cells with signaling processes
	Hypothesized Interaction Distribution [121]	$\hat{h}\left(i,\;j\right)=n^{-1}\overset{n}{\underset{i=1}{\sum }}\underset{i\neq j}{\sum }1\left(d\left(c_{i},c_{j}\right)\leq r\right)$	$\left(n-1\right)*\pi \;r^{2}$	No edge correction Mean increases with number of cells
	Empty Space Function [122]	$\hat{F}\left(r\right)=m^{-1}\overset{m}{\underset{i=1}{\sum }}1\left(r\underset{j}{\leq \min}d\left(l_{i},c_{j}\right)\leq r+\Delta r\right)e_{ij}$	$1-\exp\left(-\lambda \pi r^{2}\right)$	Summarizes much smaller scale than K, L, and M For a point process, both G and F have the same distribution Sensitive to processes with distance between point constraints [116]
	Nearest Neighbor Function [116]	$\hat{G}\left(r\right)=n^{-1}\overset{m}{\underset{i=1}{\sum }}1\left(r\underset{j}{\leq \min}d\left(c_{i},c_{j}\right)\leq r+\Delta r\right)e_{ij}$	$1-\exp\left(-\lambda \pi r^{2}\right)$	
	Hazard Empty Space Function [123] or Hazard Nearest Neighbor Function	$h_{\alpha }=\frac{d}{dr}\left(-\log\left(1-\hat{\alpha }\left(r\right)\right)\right)$	2πrλ	Interpretation similar to time to event survival analysis
	J-function [124]	$\hat{J}\left(r\right)=\frac{1-\hat{G}\left(r\right)}{1-\hat{F}\left(r\right)}$	1	Constant mean value Robust against edge corrections

P= number of pixels; $p_{i}^{j}=$ proportion of the population of cell type i in the jth area or pixel; $p_{i}=$ proportion of the population of cell type i across the entire image; n= total number of cells; $e_{ij}=$ edge correction for the ith and jth cell; $l_{i}=$ the ith randomly selected location; κ is a kernel function; $h_{\alpha }=$ hazard function of α=F or G. Blue text corresponds to spatial point processes that based on the location of cells. These methods are also referred to as second order methods. Red text corresponds to spatial point process methods that focus on distance to the nearest neighbor.

## Data Availability

The data presented in this review are available on request from the corresponding author.
